# The effect of concomitant drugs on oncological outcomes in patients treated with immunotherapy for metastatic urothelial carcinoma: a narrative review

**DOI:** 10.32604/or.2024.057278

**Published:** 2025-03-19

**Authors:** MICHELE MAFFEZZOLI, GIULIA CLAIRE GIUDICE, GIACOMO IOVANE, MARTINA MANINI, ELENA RAPACCHI, GIUSEPPE CARUSO, NICOLA SIMONI, STEFANIA FERRETTI, STEFANO PULIATTI, DAVIDE CAMPOBASSO, SEBASTIANO BUTI

**Affiliations:** 1 Medical Oncology Unit, University Hospital of Parma, Parma, 43126, Italy; 2 Department of Medicine and Surgery, University of Parma, Parma, 43126, Italy; 3 Radiotherapy Unit, University Hospital of Parma, Parma, 43126, Italy; 4 Department of Urology, University of Modena and Reggio Emilia, Modena, 41124, Italy; 5 Urology Unit, University Hospital of Parma, Parma, 43126, Italy

**Keywords:** Immune checkpoint inhibitors (ICIs), Urothelial carcinoma (UC), Concomitant drugs, Proton pump inhibitors, Antibiotics (Abs), Angiotensin-converting enzyme inhibitors (ACEIs)

## Abstract

**Background:**

immune checkpoint inhibitors (ICIs) have revolutionized the treatment of metastatic urothelial carcinoma (mUC), significantly improving survival outcomes. However, a subset of patients do not respond to ICIs, prompting research into potential predictive factors. Commonly prescribed medications such as corticosteroids, proton-pump inhibitors (PPIs), antibiotics (Abs), antihypertensives, and analgesics may influence ICI effectiveness.

**Methods:**

we conducted a literature search on PubMed to investigate the impact of concomitant medications on the outcomes of patients with mUC, treated with ICIs. We selected the most relevant studies and performed a narrative review.

**Results:**

corticosteroids, PPIs and Abs have been associated with reduced survival in ICI-treated patients, including those with mUC. In contrast, antihypertensive agents like renin-angiotensin system inhibitors and beta-blockers may enhance ICI efficacy, though evidence remains inconclusive. The impact of other medications, such as statins, metformin, and analgesics, on ICI outcomes is less clear, with some data suggesting a detrimental impact on immune response.

**Conclusions:**

this narrative review synthesizes current evidence on how concomitant medications affect outcomes in mUC patients treated with ICIs.

## Introduction

In recent years, immune checkpoint inhibitors (ICIs) targeting the programmed cell death protein-1 (PD-1) and its ligand (PD-L1) have dramatically improved survival outcomes for patients with metastatic urothelial carcinoma (mUC) [1]. Currently, ICIs are approved for use as first-line therapy in platinum-ineligible patients [2], as second-line treatment following progression on platinum-based chemotherapy [2], and as maintenance therapy after a response (or stable disease) to platinum-based chemotherapy [3]. Recently, combinations of nivolumab plus chemotherapy and pembrolizumab plus enfortumab vedotin compared to standard platinum-chemotherapy have shown to improve survival in first-line setting [4,5].

Despite these advancements, a portion of patients do not respond to ICIs, leading to extensive research aimed at identifying clinical or biological biomarkers predictive of ICI response [6]. Among the clinical factors influencing ICI effectiveness, concomitant medications have garnered increasing interest. Drug interactions and the potential anticancer effects of commonly used drugs may impact oncological outcomes of patients with several solid tumors, including urothelial carcinoma (UC), treated with ICIs [7].

Oncologic patients often have significant comorbidities and symptoms related to metastatic disease or adverse effects from therapy, thus requiring different types of concurrent drugs. Commonly prescribed medications include proton-pump inhibitors (PPIs), corticosteroids, antibiotics (Abs), anti-hypertensives, and analgesics [7].

Corticosteroids are frequently used to manage cancer-related symptoms (such as fatigue, dyspnoea, cerebral oedema, and pain), immune-related adverse events (irAEs), and autoimmune comorbidities, although they can induce an immunosuppressive state, potentially reducing ICI effectiveness. Available data suggest that steroid use before or shortly after ICI initiation is associated with poorer clinical outcomes, whereas steroid use for irAEs does not completely diminish the antitumor response of ICIs [8,9].

PPIs are among the most commonly prescribed medications in both cancer and non-cancer patients for chronic gastritis, gastric protection during treatment with steroids or non-steroidal anti inflammatory drugs and gastro-esophageal reflux. PPIs have also been linked to shortened survival outcomes in patients receiving ICIs for different solid tumors, including UC [10–14]. The mechanisms behind this influence may be mediated by a PPIs-induced reduction of gut microbiota diversity, which may ultimately impair the immune microenvironment and diminish the effectiveness of ICIs [15,16].

With a similar mechanism, the concomitant use of Abs can negatively impact survival outcomes of patients with solid tumors treated with ICIs, including those affected by advanced UC [17–21].

Recent data suggested that the concomitant use of antihypertensive drugs could affect ICI efficacy, although with controversial evidence. Specifically, angiotensin-converting enzyme inhibitors (ACEIs) and angiotensin receptor blockers (ARBs), as well as beta-blockers, have been shown to improve survival outcomes in patients with mUC undergoing ICI treatment [22–24]. However, some studies have reported no significant differences in outcomes for cancer patients receiving these drugs [25,26]. The underlying mechanisms are not fully understood. The renin-angiotensin-aldosterone system (RAS) may promote an immunosuppressive environment, therefore the inhibition of this pathway could enhance ICI efficacy [26].

Finally, acetaminophen and opioids are commonly prescribed for relieving cancer pain and fever, although it has been reported that they may act as a potential suppressor of antitumor immunity, potentially decreasing ICIs effectiveness [27,28].

This narrative review aims to summarize the available evidence regarding the impact of concomitant drugs on survival outcomes and responses in patients treated with ICIs for mUC.

## Materials and Methods

This is a narrative review. We performed a literature search on PubMed (https://pubmed.ncbi.nlm.nih.gov/) (accessed on 03 November 2024) using various combinations of the following keywords or their synonyms: ‘concomitant’ and (‘antibiotics’ or ‘proton pump inhibitors’ or ‘anti-hypertensives’ or ‘corticosteroids’ or ‘metformin’ or ‘hypoglycemics’ or ‘statin’ or ‘acetaminophen’ or ‘opioids’ or ‘drugs’ or ‘medications’) and (‘immunotherapy’ or ‘immune checkpoint inhibitors’) and (‘urothelial cancer/carcinoma’ or ‘bladder cancer/carcinoma’). We considered abstracts, reviews, metanalyses, and clinical and observational studies, and selected the most relevant works based on their level of evidence.

## Results

### Role of proton-pump inhibitors in modulating immune response

PPIs negatively impact on survival outcomes in many advanced solid tumors when administered concomitantly with ICIs [10–14] Through their direct inhibition of H^+^/K^+^-adenosine triphosphate (ATP)ase pump, which in turn reduces gastric acidity, PPIs can induce gut dysbiosis, eventually leading to a detrimental effect on immunotherapy effectiveness [12]. In fact, several studies reported that intestinal microbiota had a significant impact on immune system and ICIs response. The influence of the intestinal microbiota on anticancer immune response can vary depending on microbial species: bacteria found in ICIs responders showed different types of immune modulations, such as *Bifidobacterium fragilis*, which activated T-helper1 cells and cross-reactivity between bacterial and tumor antigens [12]; it was also observed that gut microbiota could induce cluster differentiation (CD)8+ T cell activation and promote CD4+ T cell differentiation, while reducing Treg levels [29]. The population of bacteria associated with improved response to ICIs (e.g., *Bifidobacterium sp*, *Ruminococcaceae*, *Akkermansia muciniphila* and *Alistipes sp*) was found to be decreased by PPIs treatment; on the contrary, bacteria associated with resistance to ICIs, such as oral cavity microorganisms (e.g., *Actinomyces* spp., *Rothia mucilaginosa*, *Rothia dentocariosa*) and *Bacteroidetes* and *Escherichia coli*, were increased, due to the reduction of gastric acid secretion that would normally prevent translocation and accumulation of both ingested microbial pathogens and commensal microorganisms [12]. Tomita et al. retrospectively evaluated the beneficial role of a live biotherapeutic bacterial strain (*Clostridium butyricum*, CBM588) administered in 118 patients with advanced or recurrent non-small cell lung cancer (NSCLC) treated with ICIs and concomitant PPIs [30]. The addition of CBM588 strain led to an improvement on both progression-free survival (PFS) (median PFS: 250 days *vs*. 88 days, Hazard Ratio (HR) 0.52, *p* = 0.030) and overall survival (OS) (median OS, not reached (NR) *vs*. 208 days, HR 0.42, *p* = 0.030) [30].

Other studies exploring the potential role of PPIs in affecting ICIs response showed a direct impact in influencing tumor microenvironment (TME) in many different cancer types. In fact, both *in vitro* and *in vivo* studies demonstrated that PPIs can induce mitochondrial apoptosis and impair tumor growth through inhibition of V-ATPase activity, which regulates intracellular pH homeostasis [31,32]. In addition, a translational study conducted by Gao et al. on murine models, revealed that PPIs could promote PD-L1 protein expression and stability by inducing glycogen synthase kinase-(*GSK)-3β* phosphorylation, thus enhancing ICIs response [33].

Finally, PPIs may also directly impact inflammatory responses by reducing the secretion of adhesion molecules (e.g., intercellular adhesion molecule-1 and and vascular cell adhesion molecule-1), inhibiting cytokine production (e.g., interleukin-6 (IL-6) and tumor necrosis factor-α), and facilitating immune escape by increasing the translocation of PD-L1 to the tumor cell membrane [33,34]. Other preclinical studies showed that PPIs’ interference with the nuclear factor-κB (NF-κB) pathway in gastric mucosa cells could result in lower secretion of the potent neutrophil chemoattranct, IL-8 [35]. Moreover, PPIs could contrast neutrophils’ activity by inhibiting vacuolar (v-type) H^+^ATPases which are involved in the acidification of intracellular organelles like lysosomes, thus reducing the accumulation and release of reactive oxygen species (ROS) [36].

### Role of proton-pump inhibitors in influencing survival outcomes in patients with metastatic urothelial carcinoma treated with immunotherapy

Many studies explored the impact of PPIs in patients with advanced UC treated with ICIs, Table 1.

**Table 1:** Studies evaluating concomitant proton pump inhibitors use in patients with advanced urothelial carcinoma receiving immune checkpoint inhibitors

Author	Type of study	Year	Number of patients	Type of ICI (number, %)	Treatment line (number, %)	PPI use (number, %)	Abs and PPIs (number, %)	Type of drug (number)	Use window	PFS	OS
Okuyama et al. [41]	Retrospective	2022	155	Pembrolizumab (145, 93.6%); nivolumab (6, 3.8%), atezolizumab (2, 1.3%), durvalumab (2, 1.3%)	II (155, 100.0%)	99, 63.9%	50, 50.5%	NA	30 days	14.0 *vs*. 3.6 m	50.0 *vs*. 9.1 m
										*p* = 0.002	*p* < 0.001
										HR 1.60 (95%CI 1.02*–*2.5)	HR 1.70 (95%CI 1.04*–*2.80)
										*p* = 0.041	*p* = 0.036
Hopkins et al. [39]	*Post-hoc* analysis	2020	1360	Atezolizumab (896, 65.9%) *vs*. chemotherapy (464, 34.1%)	I (429, 47.9%), II (467, 52.1%)	286, 31.9%	53, 11%	Omeprazole (179), pantoprazole (151), esomeprazole (77), lansoprazole (45), rabeprazole (13), dexlansoprazole (6)	60 days	HR 1.38 (95%CI 1.18*–*1.62)	HR 1.52 (95%CI 1.27*–*1.83)
										*p* < 0.001	*p* < 0.001
Tomisaki et al. [40]	Retrospective	2023	135	Pembrolizumab (75, 55.6%)	II (135, 100.0%)	29, 38.7%	17, 58.6%	NA	30 days	2.8 *vs*. 7.0 m	5.3 *vs*. 17.3 m
										*p* = 0.002	*p* = 0.002
Sekito et al. [42]	Retrospective	2024	404	Pembrolizumab (404, 100.0%)	II (404, 100.0%)	121, 29.9%	28, 23.1%	NA	30 days	6.4 *vs*. 9.7 m	7.8 *vs*. 12.5 m
										*p* = 0.012	*p* = 0.010
										HR 1.35 (95%CI 1.05*–*1.73)	HR 1.42 (95%CI 1.08*–*1.87)
										*p* = 0.020	*p* = 0.011
Fukuokaya et al. [43]	Retrospective	2022	227	Pembrolizumab (227, 100.0%)	II (227, 100.0%)	86, 37.9%	23, 26.7%	Vonoprazole (30), esomeprazole (22), lansoprazole (21), rabeprazole (9), omeprazole (4)	30 days	2.5 *vs*. 4.1 m	9.5 *vs*. 18.8 m
										*p* = 0.0001	*p* = 0.0002
										HR 1.7 (95%CI 1.23*–*2.35)	HR 2.02 (95%CI 1.28*–*3.18)
										*p* = 0.001	*p* = 0.003
Kunimitsu et al. [44]	Retrospective	2022	79	Pembrolizumab (79, 100.0%)	II (79, 100.0%)	34, 43.0%	NA	NA	60 days	3.5 *vs*. 5.1 m	8.2 *vs*. 11.2 m
										HR 1.63 (95%CI 0.95*–*2.80)	HR 1.36 (95%CI 0.75*–*2.42)
										*p* = 0.069	*p* = 0.296
Iida et al. [45]	Restrospective	2024	133	Pembrolizumab (133, 100.0%)	II (94, 70.7%) and III (39, 29.3%)	51, 38.3%	16, 31.4%	NA	30 days	1.8 *vs*. 4.1 m	6.1 *vs*. 13 m
										*p* < 0.001	*p* = 0.031
Fiala et al. [7]	Retrospective	2023	802	Pembrolizumab (802, 100.0%)	II (802, 100.0%)	372 (46.0%)	NA	NA	30 days	4.5 *vs*. 7.2 m	8.7 *vs*. 14.1 m
										*p* = 0.002	*p* < 0.001
Ruiz*–*Banobre et al. [46]	Retrospective	2021	119	Atezolizumab (80, 67%), pembrolizumab (29, 24%), nivolumab (7, 6%), durvalumab (3, 3%)	I (22, 18%), II (86, 72%), III and beyond (11, 10%)	54 (45%)	11 (9%)	NA	30 days	HR 1.94 (95%CI 1.22*–*3.09)	HR 1.83 (95%CI 1.11*–*3.02)
										*p* = 0.005	*p* = 0.02

Note: Abs: antibiotics, CI: confidence interval, HR: hazard ratio, ICI: immune checkpoint inhibitor, m: months, NA: not available, PFS: progression-free survival, PPI: proton pump inhibitor, OS: overall survival.

A retrospective multicenter study by Fiala et al. analyzed data regarding concomitant PPIs in a cohort of 802 patients with mUC treated with pembrolizumab: the use of PPIs had a significantly detrimental effect on PFS (4.5 *vs*. 7.2 months, *p* = 0.002) and OS (8.7 *vs*. 14.1 months, *p* < 0.001) compared to non-use, even after adjustment in a multivariate Cox analysis [7]. The same results were confirmed in another retrospective study conducted on 1360 patients with advanced mUC treated with atezolizumab within the IMvigor210 (single-arm atezolizumab trial in first-line setting) [37] and IMvigor211 (phase III randomized trial of atezolizumab *vs*. chemotherapy in first-line setting) [38,39]. Interestingly, no association between PPI use and survival outcomes were found in the participants who received chemotherapy in IMvigor211 [39]. The detrimental effect of concomitant PPIs was further reported in a multicentre study conducted on 135 patients affected by mUC and treated with pembrolizumab or paclitaxel-gemcitabine after platinum-based chemotherapy [40].

In this study, PPIs usage was associated with significantly reduced PFS and OS in patients receiving pembrolizumab, although this effect was not observed in patients treated with chemotherapy, even when corrected for possible confounding factors in a multivariate analysis [40]. Of note, the negative impact of PPIs on ICIs efficacy seems to be influenced by patients’ characteristics: in particular, it was observed that survival outcomes were significantly decreased in younger and male patients [43].

Furthermore, other studies have evaluated the association between PPIs and Abs on survival outcomes in mUC patients treated with immunotherapy [41,47]. In a retrospective study, the objective response rate (ORR) was significantly reduced in patients using both PPIs and Abs (ORR = 12%, *p* = 0.004) or either one of them (ORR = 33%, *p* = 0.010). PFS after ICI therapy was significantly reduced in the double users (median 3.0 months) than in the non-users (median 37.0 months, *p* < 0.001) or single users (median 5.8 months, *p* = 0.035). OS after ICI therapy was significantly shorter in the double users (median 6.5 months) than in the non-users (median 50 months, *p* < 0.001) or single users (median 15 months, *p* = 0.015) [41]. Similar results were described in a Japanese retrospective study [47].

Furthermore, when compared with different medications used in chronic diseases (e.g., statins, metformin) PPIs were found to be the only concomitant therapy that negatively affected survival outcomes [7,46].

Interestingly, Sekito et al. compared histamine-2 receptor antagonists (H2RAs) with PPIs in a retrospective multicentre study including 404 patients. The authors observed that the use of PPIs was a negative prognostic factor for both OS (HR = 1.42, 95% CI 1.08–1.87, *p* = 0.011) and PFS (HR = 1.35, 95% CI 1.05–1.73, *p* = 0.020), while H2RAs was not associated with survival or ORRs, thus representing a valid alternative during ICIs administration [42].

Ultimately, the detrimental effect of PPIs on survival outcomes was confirmed in multiple meta-analyses comprising different solid tumors, including advanced UC [12,48–50]. Among these metanalyses, it is worth to mention that Zhang et al. [48] and Rizzo et al. [14] have taken into account only patients with mUC. Zhang et al. analysed data from six studies involving 1980 patients with advanced UC and found that concomitant PPIs were associated with an increased risk of progression and death of 50.7% (HR: 1.507, 95% CI: 1.327–1.711, *p* < 0.001) and 58.7% (HR: 1.587, 95% CI: 1.367–1.842, *p* < 0.001), respectively [48]. Furthermore, ORR was significantly reduced in mUC patients treated ICIs and PPIs (OR: 0.503, 95% CI: 0.360–0.703, *p* < 0.001). Rizzo et al. found similar results in their meta-analysis, which included two studies encompassing a total of 1015 mUC patients [14]. Both meta-analyses showed a low level of heterogeneity (I^2^ = 7.4% and I^2^ = 0.0% for PFS, I^2^ = 37.4% and I^2^ = 0.0% for OS, I^2^ = 47.0% for ORR, respectively). Other metanalyses also provide an interesting comparison with other solid tumors: Lopes et al. observed that PPIs would negatively affect OS and PFS in patients with advanced NSCLC receinving ICIs [12]. On the other hand, Chang et al.’s work showed limited or no detrimental effect on survival outcomes in advanced renal cell carcinoma (RCC), hepatocellular carcinoma (HCC), head and neck squamous cells carcinomas and melanoma, during concomitant immunotherapy [13].

### Role of antibiotics in modulating immune response

Abs are one of the most commonly used drugs in oncological patients; especially, those affected by UC have a relatively high risk of urinary tract infections (UTI) due to urinary diversion [17]. Abs are known to have several adverse effects, including enterocolitis caused by damage to commensal gut microbiota, which plays an important role in regulating homeostasis and immune function, finally leading to a negative impact on systemic immune response [17,20]. At the same time, cancer can alter the composition of gut bacteria, which plays a role in regulating the TME and promoting immune suppression. As a result, cancer cells and self-reactive immune cells may potentially cross-react with bacterial populations [51]. Notably, the gut microbiota has gained recognition as a crucial factor influencing cancer treatment outcomes and is thought to contribute to the variability observed in patient responses to immunotherapy, especially ICIs [52]. One study reported that mice with a disruption of the microbiota exhibited inferior immune-mediated responses to medical therapies, suggesting that an intact microbiota is essential for an optimal response to cancer therapies, including chemotherapy and immunotherapy [53]. Moreover, fecal microbiota transplant (FMT) from responders (R-FMT) and nonresponders (NR-FMT) into germ-free (GF) mice confirmed the microbial modulation of antitumor immune responses. Compared with R-FMT mice, NR-FMT mice had more rapid tumor growth and poorer responses to anti-PD-1 therapy, which indicated that the gut microbiota could be a modulator of the responses to ICIs [54,55]. Abs use may impact the patient’s microbiota composition for a long time [56].

Thus, some researchers hypothesize that dysbiosis of the gut microbiota caused by Abs may be associated with ICIs resistance and have a negative impact on ICIs efficacy [57]. Several studies have demonstrated differences in the stool microbial composition in responders and non-responders to ICIs for lung, renal and melanoma patients [19]. However, the favorable gut microbiota composition and diversity that produces the most optimal response to ICI is yet to be elucidated [19]. There were some suggestions from metagenomic studies that high diversity and differential abundance of beneficial bacterial taxa in the gut, such as Ruminococcaceae, Akkermansia, Bifidobacterium, Bacteroides, Faecalibacterium, and *Akkermansia muciniphilla* may be closely linked to positive response to ICIs [18]. Their advantage arises from an active and dynamic interaction with the immune system, promoting enhanced dendritic cell maturation, improved priming, and increased accumulation of effector T-cells within the tumor microenvironment [52].

Some Abs may also have a direct impact on systemic inflammation and immune response: quinolones can lower the levels of pro-inflammatory cytokines, and macrolides can reduce the T cell response, resulting in a potential negative effect on response to ICIs [57]. These findings have led to a paradigm shift, with the gut microbiome now being regarded as a significant predictive biomarker for treatment response and a potential therapeutic target to enhance the effectiveness of immunotherapies. However, it is important to acknowledge that some studies have found no link between antibiotic use and response to ICIs, and findings from observational cohorts may be influenced by the overall health condition of patients requiring Abs use. In fact, patients who require Abs are more likely to have multiple comorbidities and moderate to severe infections [17,18]. These patients likely experience treatment interruption, with a potential impact on disease progression and poorer survival [18]. Ultimately, timing also represents a crucial element on the impact of Abs use on ICIs outcomes. In fact, the period including the month prior and the month following the start of ICIs, appeared to be the most vulnerable to Abs use, potentially for the impact of dysbiosis on the immune “priming” response [34].

With such assumptions, it becomes crucial to understand if the effects of Abs on the efficacy of ICIs is clinically relevant [17].

### Prognostic impact of antibiotics use in influencing survival outcomes in patients with metastatic urothelial carcinoma treated with immunotherapy

Several meta-analyses demonstrated that administration of Abs either before or during treatment with ICIs, was associated with shorter PFS and OS in multiple solid tumors, including NSCLC [57], RCC [57], melanoma [57] and UC [17,18,20]. Also ORR resulted significantly reduced in patients treated with ICIs and concomitant Abs [21,57,58]. Furthermore, the detrimental effect on survival outcomes was independent from the type of cancer and the type of ICI used [21,57–59].

Patients with advanced NSCLC are particularly vulnerable to receive Abs during the course of their disease or in the months leading up to their cancer diagnosis, due to the median advanced age and the smoking status [60]. A meta-analysis of 23 studies on patients with NSCLC receiving ICIs, described a detrimental effect of concomitant Abs use in terms of OS (HR 1.69, 95%CI 1.25–2.29) and PFS (HR 1.47, 95%CI 1.13–1.90) [60]. Based on this evidence, it can be hypothesized that Abs negatively affect ICI efficacy and likely contribute to the development of resistance to anti-PD-1 antibodies. However, additional research is necessary to establish a definitive causal link between antibiotic use and resistance to ICI therapy.

Due to the high risk of urinary tract and upper respiratory infections, Abs are also frequently used in RCC patients. Luo et al. demonstrated that Abs exposure was significantly associated with worse PFS and OS in RCC patients receiving ICIs; however, no significant link was found between concomitant Abs use and increased risk of disease progression [61]. In contrast, a retrospective study involving 749 patients with melanoma who received Abs compared to 1856 non-exposed patients suggested that Abs use prior to anti-PD-1 treatment was not linked to poorer outcomes, either in terms of OS or time to treatment discontinuation [62]. Different studies have evaluated the prognostic role of Abs administration specifically in patients with mUC, Table 2.

**Table 2:** Studies evaluating concomitant antibiotics or anti*–*hypertensive use in patients with advanced urothelial carcinoma receiving immune checkpoint inhibitors

Author	Type of study	Year	Number of patients	Type of ICI (number, %)	Treatment line (number, %)	Type of drug use (number, %)	Type of drug (number, %)	Use window	PFS	OS
Hopkins et al. [20]	Post*-*hoc analysis of IMvigor210 and IMvigor211	2020	896	Atezolizumab (896, 100.0%)	II (896, 100.0%)	Abs (NA)	Penicillins (87, 37.0%), quinolones (8, 37.0%), cephalosporins (84, 36.0%), glycopeptides (25, 11.0%), carbapenems (17, 7.0%), sulphonamides (17, 7.0%), macrolides (10, 4.0%), aminoglycosides (9, 4.0%), tetracyclines (7, 3.0%)	30 days prior and 30 days after ICI	IMvigor210	IMvigor210
									HR 1.24 (95% CI 1.05–1.46)	ICI in Abs *vs*. no Abs HR 1.44 (95% CI 1.19–1.73)
										IMvigor211
										ICI *vs*. CT in Abs and no Abs HR 0.95 (95% CI 0.71–1.25) *vs*. 0.73 (95% CI 0.60–0.88)
										*p* = 0.1
Ishiyama et al. [17]	Retrospective	2021	67	Pembrolizumab (67, 100.0%)	II (51, 85.7%), III and beyond (15, 25.2%)	Abs (NA)	Cephalosporins (9, 60.0%), carbapenems (3, 20.0%), quinolones (2, 13.3%), penicillins (1, 6.7%)	60 days prior and 30 days after ICI	1.1 *vs*. 8.9 m	2.3 *vs*. 19.5 m
									*p* < 0.001	*p* < 0.001
Agarwal et al. [63]	Retrospective	2019	101	Atezolizumab (52, 51.5%), pembrolizumab (39, 38.6%), durvalumab (9, 8.9%), unknown (1, 0.9%)	I (25, 24.8%), II (74, 73.2%), unknown (2, 18.2%)	Abs (54, 45.0%)	NA	30 days prior and during ICI	NA	HR 1.93 (95% CI 1.93–3.42)
										*p* = 0.024
Khan et al. [64]	Retrospective	2020	130	NA	NA	Abs (NA)	Quinolones (29, 34.0%), cephalosporins (23, 27.0%), penicillins/carbapenems (15, 18.0%), trimetropin-sulfametoxazole (12, 14%), unknown (5, 17.0%)	60 days prior or after ICI	21 *vs*. 18 weeks	51 *vs*. 61 weeks
									Abs after ICI HR 1.3 (95% CI 0.77–2.2)	Abs after ICI HR 1.62 (95% CI 0.9–2.92)
									ABS prior ICI HR 0.91 (95% CI 0.462–1.79)	Abs prior ICI HR 1.46 (95% CI 0.65–3.28)
									*p* = 0.8	*p* = 0.6
Jain et al. [22]	Retrospective	2021	Cohort 1: 178	Atezolizumab (83, 46.6%),	I (Cohort 1: 25, 14.0%; Cohort 2: 42, 41.5%)	ACE inhibitor: Cohort 1: 33, 18.5%	NA	Ongoing from ICI start	NA	Cohort 1
			Cohort 2: 101	Pembrolizumab (79, 44.4%), other (16, 9.0%)						30.4 m (95% CI, 14.5–63.0) *vs*. 13.0 m (95% CI, 7.6–17.2)
					II (Cohort 1: 153, 86%; Cohort 2: 59, 58.4%)	Cohort 2: 22, 21.8%				HR 0.37 (95% CI, 0.12–1.15)
										*p* = 0.086
										Cohort 2
										NR *vs*. 19.7 m (95% CI, 11*–*NE)
										HR 0.37 (95% CI 0.12–1.15)
										*p* = 0.086

Note: Abs: antibiotics, ACE: angiotensin converting enzyme, CI: confidence interval, HR: hazard ratio, ICI: immune checkpoint inhibitor, m: months, NA: not available, NE: not evaluated, NR: not reached, PFS: progression-free survival, OS: overall survival.

Febriyanto et al. conducted a metanalysis on thirteen non-randomized studies, including a total of 5095 patients with mUC treated with ICIs, of which 1434 (28%) received Abs. The pooled HRs for OS and PFS in those who received Abs were 1.45 [95% CI 1.25−1.68] and 1.40 [95% CI 1.05−1.87], respectively, compared to those who did not receive Abs [18]. Again, the type of ICI used did not influence the effect of Abs on OS and PFS [18].

The optimal time window for Abs use remains a critical point to explore, in fact the time of Abs exposure might impact ICIs effectiveness [18,21,65]. The time it takes to restore the gut microbiota composition and mount an effective antitumoral immune response following antibiotic use remains unclear [18]. Abs appear to influence ICIs outcomes even after their withdrawal, with a possible deleterious effect over a long period [65]. Especially, Abs use in the 42 days before starting ICIs appears to have the most detrimental impact on outcome [17]. A previous study by Pinato et al. showed that Abs treatment administered within 30 days prior to ICI therapy was associated with significantly worse OS, but not with concurrent ICI therapy [66]. Of note, this was the only prospective study reported in literature, included 196 patients with different tumor types [66]. In another study by Khan et al., the maximal negative impact on the effectiveness of ICIs occurred when Abs were used in the first six weeks after initiating ICI [64]. Even the metanalyses by Huang et al. [67] and Yu et al. [21] showed an impact on the efficacy of ICIs within before or after two months, while a better PFS and OS was demonstrated concurrently with ICIs therapy [57].

The type of Abs used seems not to influence survival outcomes, although the use of broad-spectrum Abs was strongly associated with poor PFS [59].

Some retrospective studies have also studied the impact of Abs specifically on patients with mUC receiving ICIs. In 2020, Hopkins et al. performed a *post-hoc* analysis of IMvigor210 and IMvigor211 to study the association between Abs use within 30 days before and after ICIs initiation, and survival [20]. Interestingly, Abs use was associated with worse PFS and OS in patients treated with atezolizumab, but not with chemotherapy [20]. In 2021, Ishiyama et al. confirmed these findings in a cohort of 67 patients treated with pembrolizumab [17]. Of note, patients who were administrated Abs were also less likely to achieve response or disease control [17]. Accordingly, Agarwal et al. showed that concurrent Abs can influence outcomes and response in patients with mUC receiving both anti-PD1 and anti-PD-L1 agents [63].

Conversely, Khan et al. involved 130 patients with mUC treated with different type of ICIs. This was the only study showing that Abs use within 60 days before or after ICI initiation did not significantly impact OS and PFS [68].

### Role of antihypertensive drugs in modulating immune response

Pre-clinical evidence highlighted a possible role of the RAS as a pro-inflammatory modulator in the TME [69–71]. Specifically, the RAS was associated with an enhanced immunosuppressive environment through upregulation of PD-L1 expression and the presence of immunoregulatory cells such as tumor-associated macrophages, myeloid-derived suppressor cells, and cancer-associated fibroblasts [72,73]. From a biological perspective, pre-clinical studies suggested that the inhibition of the RAS may down regulate transforming growth factor (TGF)-β, a transcription factor, mainly active in TME stromal and fibroblasts cells. TGF-β is a component of multiple signalling pathways, regulating proliferation, apoptosis, invasion, migration, immunosuppression, chemo-resistance and progression [74]. Additional evidence underline RAS ability of promoting angiogenesis via vascular endothelial growth factor (VEGF) synthesis [75]. The RAS inhibition may consequently have anti-fibrotic effects, decrease stromal collagen I, impact tumor perfusion and drug delivery [69,76]. Accordingly, various evidence proposed that a TGF-β increase was related to resistance to ICI [77], thus, inhibiting the RAS may enhance ICI responses [78]. Furthermore, pre-clinical evidence proposed a possible role of RAS inhibition in inducing a pro-inflammatory TME [69–71,79]. In details, RAS appears to enhance the TME PD-L1 expression, the immunosuppressive phenotype of tumor associated macrophages (TAMs) and myeloid-deprived suppressive cells (MDSCs), mainly through the production of immunosuppressive chemokine [72,73,79]. This hypothesis was also supported by evidence that ARBs could reverse ICI resistance in mouse models [79]. Nevertheless, the data remain controversial.

β-blockers are another widely used class of antihypertensive drugs, whose activity appears to be related to an immunosuppressive TME. Specifically, β-adrenergic receptors, which are present on lymphoid organs and immune cells [80], can down regulate T-cell proliferation and cytotoxicity, and stimulate immunosuppressive regulatory T cells [81]. The inhibition of β-adrenergic receptors enhanced CD8+ T cells recruitment and activation and PD-1 expression [82]. Additionally, pre-clinical data suggested a role of β-blockers in inhibiting tumor growth, when used in combination with ICIs [82,83].

### Do antihypertensive drugs impact on ICIs effectiveness?

A retrospective study on patients affected by solid tumors receiving ICIs, including bladder, ovarian and prostate cancer, described improvements in terms of ORR, CR rate and OS, in those with concomitant ACEIs or ARBs [84]. Similarly, an observational analysis on advanced RCC correlated longer OS with RAS inhibitors use [85]. In contrast, a retrospective analysis identified a direct correlation between the use of ACE inhibitors and poorer ICI outcomes in patients with NSCLC [86]. Ultimately, retrospective evidence on patients with NSCLC [87] or on a larger cohort of patients with solid tumors (NSCLC, RCC or UC) receiving ICIs, highlighted no correlation between outcomes and RAS inhibitors use [26].

Modest data are available for patients with mUC. A retrospective analysis of 279 patients with UC treated with ICIs and concomitant ARBs or ACE inhibitors found a positive association with OS (Table 2**)** [22]. Additionally, a pooled analysis of seven studies was conducted, including 2539 patients receiving atezolizumab and concomitant antihypertensive treatments (RAS inhibitors, ACE inhibitors, ARBs, direct renin inhibitors, and beta blockers), about 35% of whom affected by UC (n = 888) [26]. This analysis noted no association between the use of anti-hypertensives and oncological outcomes in terms of OS and PFS [26]. The only statistically significant finding was a worse PFS associated with the concomitant use of ARBs (adjusted HR 1.16, 95% CI 1.01–1.33, *p* = 0.038) [26]. In order to further investigate the impact of RAS inhibitors on ICI outcomes, a meta-analysis of twelve retrospective or integrated *post hoc* studies was led. This meta-analysis included a total of 11,739 patients with UC, RCC, melanoma, and NSCLC, with 12%–58% of them receiving concomitant RAS inhibitors [24]. Significant heterogeneity was observed among the studies (I^2^ = 52.1%, *p* = 0.010 for OS and I^2^ = 61.0%, *p* = 0.012 for PFS). The metanalysis found a significantly better OS [pooled HR 0.85 (95% CI, 0.75–0.96; *p* = 0.009)] and a trend toward better PFS [pooled HR 0.91 (95% CI, 0.76–1.09; *p* = 0.296)] in RAS inhibitors users [24]. Furthermore, the subgroup analysis of UC patients confirmed a statistically significant benefit for OS (HR 0.53; 95% CI, 0.31–0.89; *p* = 0.018) but not for PFS [24]. The association was found only with the simultaneous use of antihypertensives, with no effect reported on the incidence of irAEs [24].

Regarding the impact of concomitant β-blockers use on ICI outcomes, the evidence remains controversial. Two similar observational studies on melanoma and NSCLC, suggested a potential survival benefit in patients treated with ICIs and concomitant β-blockers [83,88]. However this evidence was not confirmed from other retrospective data. For instance, a detrimental effect of concomitant β-blockers use in NSCLC patients receiving ICIs, was described [87]. Another a retrospective analysis was conducted involving 339 patients with solid tumors (melanoma, NSCLC, hepatocellular carcinoma, and UC) who were receiving ICIs [23]. This analysis compared patients treated with β-blockers (n = 109%, 32%) to those without concurrent β-blocker treatment. The study found a positive correlation between concurrent β-blockers and disease control rate (62% *vs*. 39%, odds ratio (OR) 2.79, *p* < 0.001) in the overall population, and with OS in UC patients (HR 0.24, *p* = 0.003) [23]. Additionally, a phase I trial testing the association of pembrolizumab and propranolol, a β-blocker, showed promising results, with ORR of 78% in patients affected by melanoma [89]. Ultimately, a systematic review and metanalysis of nine studies was conducted. This analysis evaluated 1364 patients with melanoma, NSCLC, RCC, and UC. No significant association between β-blockers use and either OS or PFS was found [pooled HR 0.99 for OS and 0.97 for PFS] [25].

### Additional medications

Many other drugs are being studied for their potential role when used in combination with immunotherapy treatments.

The negative impact of corticosteroid use on ICIs responses is well-known [90] and was confirmed in a retrospective analysis of 8870 patients, 47.8% of whom were treated with concomitant steroids. In the subgroup of patients with UC (10.8%), corticosteroid use was associated with worse early progressive disease (OR 1.49 (1.11–2.01), *p* = 0.01) and OS (1.43 (1.19–1.72) *p* < 0.0001) [28]. Data suggest that steroid use before or shortly after ICI initiation is associated with poorer clinical outcomes [8,9,28,90].

The role of statins and metformin, two common concomitant drugs, has recently been explored in patients treated with ICIs [7].

*In vitro* evidence has shown that statins can inhibit tumor cell growth and invasion [91,92]. Specifically, the inhibition of 3-hydroxy-3-methylglutaryl coenzyme A (HMG-CoA) reductase, and the following depletion of isoprenoids, appear to prevent the G1 to S phase transition [93]. Furthermore, statins might induce cell apoptosis through the regulation of pro-apoptotic and anti-apoptotic factors [93]. Ultimately, statins were able to inhibit the metastatic ability of breast cancer cell [94]. A meta-analysis of 27 studies, including 163,005 patients with advanced-stage solid tumors, mainly lung, pancreatic and ovarian cancers, described longer OS (HR 0.74), CSS (HR 0.74) and PFS (HR 0.76), in concomitant statins users [95]. In relation to ICIs, statins have demonstrated a role in regulating T-cell activity and migration, antigen presentation, and cytokine production [96]. A retrospective study of 1510 patients with non-muscle invasive bladder cancer highlighted a potential impact of statins in reducing recurrence rates [97]. Furthermore, retrospective evidence of 2602 patients with NMIBC receiving intravesical bacille Calmette-Guérin described longer OS and CSS, with concomitant statins use [98]. A retrospective analysis of 219 patients with RCC receiving nivolumab showed longer OS and PFS in statin users (*p* = 0.017 and *p* = 0.013, respectively) [92]; accordingly, some evidence proposed a potential adjuvant benefit of concomitant statins, in terms of OS, in patients with HCC [99], NSCLC [100]. Statins were also related to better ORR, longer PFS and OS, in a retrospective cohort of patients with thoracic cancer (82 with malignant pleural mesothelioma and 179 with NSCLC) treated with PD-1 inhibitors [96]. However, this benefit was not observed in a population of patients with UC treated with ICIs [7,101].

A potential role of metformin in controlling cancer cell growth, both direct and indirect, and enhancing ICI responses has also been described [91]. Metformin may directly inhibit pathway related to cancer invasion and migration, including AMP-activated protein kinase (AMPK), epithelial-mesenchymal transition (EMT), VEGF and mammalian target of rapamycin (mTOR) [102], thus inhibiting cancer cell progression [103]. Moreover, its anti-inflammatory and hypoglycemic effects, represent protective factors against cancer development [104]. On a biological level, metformin might enhance natural killer (NK) and cytotoxic T cells, and decrease regolatory T cells and myeloid-derived suppressor cells (MDSCs) [105]. Intriguingly, pre-clinical evidence suggest metformin might inhibit both PD-1 and PD-L1, in the TIME, increasing ICIs responses [106,107]. However, the evidence remains controversial. Some observational studies on cohorts of patients with solid cancer suggested a potential benefit of concomitant ICIs and metformin, in terms of ORR, PFS and OS [91,108,109], whereas various other retrospective analyses did not confirm this evidence, neither in a cohort of metastatic RCC [110], nor in melanoma patients treated in adjuvant setting [111]. Conversely, metformin use appeared to be detrimental in a cohort of HCC patients, receiving atezolizumab and bevacizumab [112]. Regarding UC, in the real-world ARON-2 study, data from 802 patients with UC treated with ICIs were collected. The concomitant use of metformin (12% of patients) did not show any influence on ICI outcomes (PFS: 7.1 (95%CI 3.7–12.0) *vs*. 6.2 (95%CI 5.0–6.9) months, *p* = 0.630; OS: 12.4 (95%CI 7.8–16.0) *vs*. 10.5 (95%CI 9.0–13.3) months, *p* = 0.896) [7].

Recent data suggest a negative impact of acetaminophen on oncological response to ICIs. Specifically, pre-clinical studies have shown inhibition of immune cell proliferation and activity [113], as well as a decrease in interferon-induced responses [27]; this evidence is supported by recent data showing reduced vaccination response in patients receiving acetaminophen [114]. A retrospective analysis of the phase III trial CheckMate 025, which investigated nivolumab in patients with advanced RCC, noted that detectable levels of acetaminophen in the plasma were associated with significantly worse OS [27]. In the same study, this tendency was further confirmed in a broader population of patients with solid tumors, including those with UC (5%), receiving ICIs. Detectable levels of acetaminophen were again associated with worse OS (*p* < 0.0001) and PFS (*p* = 0.009) [27].

Opioids also appear to have a role in modulating ICIs response, worsening ICIs outcomes [115]. The biological rationale may lay in their immune suppression effect through T-cell modulation [116] and NK inhibition [117]. A real-world analysis of 8870 patients receiving ICIs, 10.8% of whom were affected by UC, highlighted the negative impact of opioids on both early progressive disease (OR 2.80; 95%CI 2.07–3.83) and OS (HR 1.68 (1.39–2.03) *p* < 0.0001) [28].

## Discussion

Searching for clinical and biological biomarkers that can predict the response to ICIs is an open issue in the oncology field. Even more challenging is identifying simple and routine factors that can be used in daily clinical practice. Patients with mUC frequently have comorbidities or disease- and therapy-related symptoms that require pharmacological intervention. Concomitant drugs are often prescribed without considering their potential influence on the effectiveness of ICIs, although recent evidence has shown that commonly used drugs may affect oncological outcomes. Hence, the influence of concomitant drugs on immunotherapy has become an area of emerging interest. Among commonly prescribed drugs, corticosteroids, PPIs, Abs, antihypertensives, and analgesics have been shown to impact the prognosis of patients treated with ICIs.

In this review, we have analyzed the impact of the aforementioned medications on the survival of patients with cancer treated with ICIs, particularly mUC. The negative impact on PFS and OS of concomitant corticosteroids, PPIs and Abs has been widely demonstrated by different retrospective studies, and confirmed by several metanalyses. Of note, some *post-hoc* analyses of prospective studies strengthened this evidence for PPIs and Abs, although only regarding patients receiving atezolizumab, which is no more recommended by regulatory agencies in this setting [20,39]. Interestingly, as already mentioned some studies reported a differential impact of concomitant PPIs and Abs between ICIs and chemotherapy, suggesting a possible predictive role [20,39,40].

The mechanism behind this effect can be mediated by the influence of PPIs and Abs on the gut microbiota. Data suggest that gut microbes may impact antitumor immunity via several mechanisms, including interaction of microbial components or products with antigen-presenting cells (APCs) and innate effectors such as Toll-like receptors, which help prime an adaptive immune response; induction of cytokine production by APCs or lymphocytes; and local or distant effects of microbial metabolites [40]. PPIs and Abs can influence the composition of the gut microbiota promoting the growth of typically oral bacteria, diminishing gut microbiota diversity and impairing the immune environment [15,16,30,57]. Recently, additional interest is emerging on intratumoral microbiota, as shown by evidence linking intratumoral Escherichia with improved survival in NSCLC patients treated with single-agent ICIs [118].

In light of this, modulation of gut and intratumoral microbiota could be an area of promising research. Intriguingly, clinical studies have reported that fecal microbiota transplantation using stool collected from ICIs responders, allows patients to overcome resistance to ICIs [32,41,44,46]. However, it is relevant to take into account that several mechanisms can alter the gut microbiota, including irAEs, such as diarrhoea, which have been cited as a positive predictor of survival in patients undergoing ICIs [17].

Differently from PPIs and Abs, ACEIs and ARBs, as well as beta-blockers, have shown to improve survival outcomes of patients with mUC undergoing ICIs, while the effect of concomitant administration of other drugs, such as statins and metformin, is not yet fully understood. Both of them can interact with the immune system, although their impact on oncological outcomes remains controversial.

Finally, analgesic drugs are frequently used in oncological patients. Interestingly, acetaminophen and opioids have been shown to reduce the immune response, thus worsening the survival of patients with solid tumors treated with ICIs. However, patients receiving analgesics for major pain might be in worse condition than other patients, thus influencing the prognosis. It is worth noting that the studies we reported are limited by possible confounding factors, among the others, the different populations, type of ICI used, and polypharmacotherapy.

Moreover, we described the impact on ICIs outcomes for each class of drugs separately. In this light, some authors have evaluated the prognostic role of polytherapy (PPIs, Abs and corticosteroids), developing and validating a “drug score” in patients with NSCLC treated with ICIs [119]. Recently, this score has been validated on a cohort of patients with mUC treated with ICIs, demonstrating that a higher number of concomitant drugs was associated with worse response and survival [47].

The mechanisms underlying the interaction between concomitant drugs and immunotherapy remain hypothetical and warrant further validation with preclinical studies and possibly with prospective trials. In recent years, considerable efforts have been made in this direction.

With respect to the possible influence of PPIs and Abs on the gut microbiota and the efficacy of immunotherapy, various mouse models have provided insights. These studies demonstrated that the efficacy of anti-CTLA4 was initially suppressed by Abs but subsequently restored through fecal transplantation or by directly feeding mice with Bacteroides isolates or probiotics [54,120].

This approach was later applied in several human studies. In the TACITO trial, patients with mRCC receiving immunotherapy combinations were randomized to receive either placebo or a fecal transplant from an immunotherapy responder [121]. In other trials, patients undergoing nivolumab and ipilimumab or cabozantinib treatment for mRCC were randomized to receive either CBM or placebo, with significant improvements in oncological outcomes [122,123].

A different approach has been used by Medik et al., who reported favorable outcomes in mice treated with anti-CTLA-4 ICI and metronidazole [124]. Using antibiotics to modulate immune responses through microbiota alterations could be a promising strategy for enhancing ICI efficacy [125]: a phase II trial led by Monge et al. is currently assessing the combination of nivolumab, oral vancomycin, and tadalafil in hepatocellular carcinoma (HCC) and liver-dominant metastatic digestive cancers (NCT03785210) [126].

Many efforts have been made also to understand the interaction between other compounds and immunotherapy. Metformin have shown to lead to the degradation of membrane PD-L1 in breast tumor mouse models as well as in patient samples, and to enhance PD-1 inhibitor efficacy in lung cancer [127,128]. Phase I and II trials are currently assessing the potential of metformin in combination with anti-PD-1 and anti-PD-L1 therapies for various cancers, including melanoma (NCT04114136), NSCLC (NCT03048500), and colorectal cancer (NCT03800602) [129,130].

A phase I study by Gandhi et al. established a dosing regimen of propranolol with pembrolizumab in melanoma patients, demonstrating satisfactory safety [89]. Ongoing phase II trials are further exploring the positive effects of beta-blockers in cancers such as triple-negative breast cancer (NCT05741164), UC (NCT04848519), and melanoma (NCT03384836, NCT05968690).

In another study, Chauhan et al. developed a tumor-selective ARB, which enhanced ICI efficacy in animal models without lowering blood pressure [74]. However, prospective clinical studies are needed to confirm these findings and establish clinical benefits.

Hopefully, the ongoing trials will expand our understanding of this pivotal field and provide some guidance for physicians’ choices in everyday practice.

A key limitation of our work is that it is not a systematic review, meaning that only the most relevant studies were included, rather than considering all available evidence. Additionally, the majority of the data analyzed are from retrospective studies, which are subject to inherent biases like selection bias, confounding factors, and incomplete information. These issues restrict the ability to draw firm conclusions regarding causality. The heterogeneity across studies, with differences in methodologies, patient populations, and treatment protocols, further complicates the consistent interpretation of results and their application to specific clinical scenarios. Moreover, the lack of randomized controlled trials (RCTs) specifically designed to assess the impact of concomitant drugs on ICI outcomes weakens the overall strength of the findings, as RCTs are considered the gold standard for establishing a cause-effect relationship. Lastly, unmeasured confounding factors, such as patient comorbidities and disease severity, may account for some of the observed associations, as these factors were not uniformly reported in the studies analyzed.

In conclusion, considering all the evidences available regarding the prognostic significance of concomitant drugs in patients receiving immunotherapy, it could be state that medications should be prescribed with caution and only when clinically necessary, especially before the initiation of ICI therapy.

## Data Availability

Data sharing not applicable to this article as no datasets were generated or analyzed during the current study.
